# The Contribution of Implementation Evaluation to the Field of Public Health

**DOI:** 10.5888/pcd20.230323

**Published:** 2023-11-02

**Authors:** Tamara Vehige Calise, Antonio J. Gardner

**Affiliations:** 1 JSI Research & Training Institute, Inc, Boston, Massachusetts; 2 The University of Alabama, Department of Community Medicine and Population Health, Tuscaloosa, Alabama

## Introduction

Chronic diseases such as heart disease, diabetes, and cancer are the leading causes of death and disability in the United States, contributing largely to the $4.1 trillion in health care costs spent per year across the nation (1). Once overlooked or considered as secondary influences on chronic diseases, social factors — education, lifestyles, living situations, financial conditions, cultural traditions, and governmental policies, among others — are now acknowledged as major contributors to health, affecting individuals, groups, and communities in positive and negative ways (2). Furthermore, these factors interact, influence, modify, and enable or constrain health interventions and program implementation across different settings. Many modern-day efforts work to change conditions, or the context within which health is produced, and may have multilevel and multidirectional interventions that perform independently and interdependently. Subsequently, the outcomes are the product of the complex interventions as well as the contexts in which they develop.

Judgements about strategies that address the social factors have traditionally been guided by randomized controlled trials (RCTs) — the “gold standard” — in identifying effective interventions (3). However, adjusting the influence of contextual factors as causal effects or controlling for these conditions in an attempt to reduce bias is nearly impossible, especially when “real life” changes in unpredictable and variable ways (4). Accordingly, there is concern that complex interventions deemed effective by RCTs may not reduce health inequities and, in fact, could widen them (5). The more we embrace diverse opportunities for ongoing learning and thoughtful conduct, appraisal, and synthesis of information used to generate evidence, the more effective we will be in addressing public health complexity rooted in effecting change (6).

## Purpose of the Special Collection

*Preventing Chronic Disease* (PCD), a peer-reviewed public health journal sponsored by the Centers for Disease Control and Prevention (CDC), promotes dialogue on the implementation and adaptation of evidence and practical experience to address inequities and improve population health (7). This special collection features 9 implementation evaluation (IE) articles — a type of article that PCD publishes — in which authors describe implementation and adaptations of interventions across a range of chronic disease risk factors that have been implemented and evaluated in real-world settings (8).

Dissemination and implementation models, theories, and frameworks are important in helping to understand context, understand how interventions work, and provide generalizable knowledge (9). Noticeable in this special collection is the use of frameworks, as well as the mixed-methods approaches to evaluation, and the alignment of work to theory. Whooten et al (10) and Harden et al (11) used RE-AIM (12) to systematically address the gap between research and practice. Although authors described physical activity (PA) programs and used elements of RE-AIM as metrics guiding their work, the approaches were different. Whooten et al (10) performed an exploratory concurrent-nested, mixed-methods evaluation of a preexisting before-school PA program. The authors highlighted adaptability and differences in implementation across the participating schools. Harden et al (11) documented adaptations made to an older adult PA program so that all audiences had access to relevant information that informs decision-making processes for training, delivery, and participation at the administrator, instructor, and participant levels. The authors presented contextual factors and processes that may be seen in the chronic disease morbidity and mortality reports more distally. Perry et al (13), who also reported on a PA program (among cancer survivors), used the Interactive Systems Framework for Dissemination and Implementation (14), which has similar elements to RE-AIM, to describe the context and processes that helped organizations implement the program.

Other authors contributed to the knowledge base by exploring specific factors of context such as partnerships, community readiness, and implementation strategies. Calancie et al (15) acknowledged the layers of complexity to prevent childhood obesity by describing a coalition approach driven by the Stakeholder-Driven Community Diffusion theory (16,17) for implementing, assessing, and analyzing collaborative efforts. The authors not only narrate the ways they assessed changes in coalition member knowledge and understanding of the problem and solutions, but they also provide details on how data were used to generate and implement action within their community. Linabarger et al (18) also presented an approach to unpack the role of collaboration in the development and implementation of dental care. The article shares insight on the use of mixed-methods evaluation to assess collaboration between the chronic disease and oral health programs of state departments to collaboratively develop and implement joint projects. The evaluation identifies many factors that facilitated collaboration including investing in relationships, creating a collaborative norm, and meeting and communicating frequently, which could be applicable in other public health areas. Long et al (19) spoke of collaboration and capacity building between academics and school staff. Although more details would be helpful to understand implementation and the specific adjustments made from evaluation input, the article demonstrated the utility of ongoing data collection and dissemination to ensure the sustainability of a complex environmental strategy to reduce sodium intake in school lunches in a large district.

Golden et al (20) described community readiness as a contextual factor to implement a pediatric weight management program in medically underserved areas and shared lessons learned on potential barriers and facilitators in communities that could affect implementation efforts. Leeman et al (21) presented methods used to assess implementation of quality improvement coaching for improving human papillomavirus vaccination coverage, part of an RCT, in an effort to identify variations, including implementation and contextual factors, across 3 states.

Finally, the article by Maxwell et al (22) is a good example of the differences in uptake, implementation, integration, and sustainability of interventions proven effective in increasing colorectal cancer screening (CCS). The authors looked at implementation across 355 clinics partnering with the Colorectal Cancer Control Program and suggested that both technical and financial support, and the ability to integrate 6 of 8 strategies into electronic health records, may be key to implementation. They also indicated that clinics may require even more support and encouragement to add 2 of the evidence-based interventions into their practice. Maxwell and her colleagues also reported that one of the evidence-based strategies is uniquely suited to reduce cancer disparities and may be of greater interest to clinics that serve populations with substantial barriers to CCS. These findings, in addition to the other studies published on this project, provide insight on context and may guide clinics in implementing or adapting approaches in their own settings.

## Implications for Public Health

Communicating the variation in implementation and effectiveness, as well as understanding the applicability of findings from one context to another, could help decision makers best use their resources to address the major contributors of chronic disease among their specific populations and across their communities. The work described in these articles focuses on processes, implementation within specific contexts, and contextual factors. Although this information is useful in its own right, we highlight 2 aspects that may improve the future reporting of IEs. First, although PCD has a checklist with approximately 40 items for authors to consider as they prepare their manuscripts (8), the level of detail presented on context and the consistency describing the intervention and implementation varied across the 9 articles. For example, several articles reported implementation adaptations and illustrated various changes, which may help those interested in the intervention understand context in terms of the organizational resources and community environment, as well as other factors.

Second, there has been a growing recognition of the importance of establishing guidance for reporting IEs, and the interactions between an intervention and its contexts (23). Checklists such as the Consolidated Standards of Reporting Trials (CONSORT) for RCTs and the Transparent Reporting of Evaluations with Nonrandomized Designs (TREND) were developed to help authors report in a consistent, transparent, and complete manner (24,25). Although these are useful contributions, a commonly used best practice on criteria to assess IEs or how context should be considered and reported does not exist, which may explain the variations in this series and the appraisal of studies against criteria not suitable for this type of evaluation.

Authors reported the limitations of their reported IEs, a standard dissemination best practice. However, several authors used efficacy and effectiveness study criteria. For example, Harden et al (11) acknowledged the importance of an iterative cycle like assess, plan, do, evaluate, and report; the desire to disseminate information so that audiences can make informed decisions; and the unpredictable timeline associated with the process. They explained that efficacy trials are not necessary if an adaptation does not threaten outcomes, yet stated their study was limited by the fact that randomization or causation could not be explored.

## Conclusion

Describing interventions and context is difficult given the possibilities and level of detail needed for those not directly affiliated to understand (26), but progress has been made. Criteria outlining the intervention and contextual categories, levels, or domains with which authors should judge their work and discuss when reporting evidence from IEs may be a great contribution to the field of public health. Consistency and details will make the evidence more useful to decision makers interested in implementing, adapting, sustaining, transferring, and scaling up interventions suited to address today’s complex public health in their respective communities.
